# Postmortem bioelectrical impedance analysis: Exploratory Assessment of phase angle behavior across different postmortem intervals

**DOI:** 10.1007/s00414-026-03863-5

**Published:** 2026-06-08

**Authors:** Matteo Antonio Sacco, Maria Cristina Verrina, Ennio Avolio, Sabrina Raffaele, Saverio Gualtieri, Gioele Grimaldi, Maria Daniela Monterossi, Chiara Caruso, Mattia Solano, Roberto Raffaele, Isabella Aquila

**Affiliations:** 1 Institute of Legal Medicine, Department of Medical and Surgical Sciences, Magna Graecia University, Catanzaro, 88100 Italy; 2 University of Calabria, Cosenza, 87100 Italy; 3 Health Center Srl, Cosenza, 87100 Italy

**Keywords:** Postmortem interval, Bioelectrical impedance analysis, Phase angle, Time since death

## Abstract

The estimation of the postmortem interval (PMI) remains a major challenge in forensic practice, particularly beyond the early postmortem phase. Bioelectrical Impedance Analysis (BIA) is a non-invasive technique widely used in clinical medicine to assess tissue electrical properties, but its application in forensic thanatochronology is still limited. This pilot study aimed to evaluate the feasibility of postmortem BIA and to investigate the relationship between PMI, body mass index (BMI), and bioelectrical parameters in a series of human cadavers. BIA measurements were performed in 29 deceased individuals using a single-frequency analyzer, recording resistance, reactance, impedance, and phase angle. The postmortem interval ranged from 1.60 to 192.87 h. Thirteen cases underwent forensic autopsy, and in ten of these subjects BIA measurements were repeated after autopsy to assess the impact of invasive procedures. Associations between PMI, BMI, and bioelectrical variables were explored using correlation analysis and exploratory regression modeling. Among the evaluated parameters, phase angle showed a moderate positive correlation with PMI (r = + 0.46), *showing a tendency toward higher values with increasing postmortem interval*. Reactance demonstrated a weak positive correlation with PMI (r = + 0.25), whereas resistance and impedance showed no meaningful association with postmortem time. No significant correlation was observed between BMI and PMI. Exploratory regression analysis indicated that the temporal behavior of phase angle was better described by a log-linear model than by a simple linear relationship, although substantial inter-individual variability remained. In all cases with paired measurements, phase angle values increased after autopsy, suggesting a systematic effect of anatomical disruption and fluid redistribution. These findings demonstrate that BIA is technically applicable in the postmortem setting and that phase angle exhibited measurable but highly variable time-related changes after death. However, the influence of autopsy procedures and the magnitude of residual variability indicate that phase angle may provide limited supplementary temporal information but cannot currently be considered a reliable PMI estimation tool. Further studies with larger cohorts and standardized protocols are required to clarify its forensic applicability.

## Introduction

The estimation of the postmortem interval (PMI) represents a cornerstone of forensic investigation and continues to be one of the most methodologically complex and scientifically challenging tasks in legal medicine [1–3]. Despite decades of research and the development of multiple thanatochronological approaches, the determination of time since death remains burdened by intrinsic biological variability, environmental interference, and methodological constraints that limit both accuracy and reproducibility [2–4]. Traditional methods based on early postmortem phenomena, including algor mortis, livor mortis, and rigor mortis, are primarily applicable within narrow temporal windows and are significantly influenced by ambient temperature, body habitus, clothing, and pre-existing pathological conditions. Similarly, biochemical, molecular, and entomological techniques, although valuable in specific contexts, often require specialized expertise, extended processing times, or assumptions that restrict their routine forensic applicability [5].

In this framework, there is a growing demand for objective, quantitative, and operator-independent methods capable of capturing postmortem biological changes through measurable physical parameters. Instrumental approaches that rely on standardized acquisition protocols and yield reproducible numerical outputs are particularly appealing, as they offer the potential to reduce subjective interpretation and inter-observer variability. Among these techniques, Bioelectrical Impedance Analysis (BIA) has emerged in clinical medicine as a robust and extensively validated tool for the assessment of body composition, hydration status, and cellular integrity, yet its application in forensic thanatochronology remains largely unexplored [6, 7].

BIA is based on the transmission of a low-intensity alternating electrical current through biological tissues and the measurement of the resulting opposition to current flow, which is determined by the conductive and capacitive properties of the body [8, 9]. Electrical resistance reflects the conductive behavior of ionic solutions, primarily influenced by total body water and electrolyte distribution, whereas reactance arises from the capacitive effect of cell membranes and tissue interfaces, which temporarily store electrical charge. The combination of these two parameters yields impedance, while their angular relationship is expressed as the phase angle. In vivo, phase angle is widely regarded as an indirect marker of cellular health and membrane integrity, with lower values typically associated with cell membrane breakdown, fluid imbalance, and poor clinical outcomes.

The postmortem environment, however, differs fundamentally from the living state, as it is characterized by the cessation of active cellular regulation and the predominance of passive physicochemical processes. Following death, progressive loss of membrane permeability control, collapse of transmembrane ion gradients, redistribution of intra- and extracellular fluids, and early autolytic phenomena are expected to alter tissue conductivity and capacitance in a time-dependent manner. These processes, which evolve continuously during the postmortem interval, provide a theoretical basis for the use of bioelectrical measurements as potential indicators of PMI. Nevertheless, the directionality and temporal behavior of individual electrical parameters after death cannot be directly inferred from in vivo models and require dedicated experimental investigation.

Preliminary forensic studies have suggested that bioelectrical parameters may exhibit systematic variations in relation to PMI, indicating that BIA may provide measurable postmortem electrical changes whose forensic significance remains uncertain [6, 7]. However, available evidence remains limited, and several critical aspects have yet to be clarified. In particular, the behavior of the phase angle in the postmortem period appears to diverge from established clinical patterns, raising questions regarding the underlying mechanisms governing postmortem electrical changes [10, 11]. Additionally, the potential influence of confounding factors such as body mass index (BMI), cause of death, comorbidities, and invasive postmortem procedures has not been comprehensively evaluated, hindering the development of standardized interpretative models.

BMI was explored as a potential source of inter-individual variability in bioelectrical measurements. However, given the exploratory nature of the study, no specific assumptions were made regarding its relationship with postmortem interval.

Against this background, the present study aims to provide *an* experimental evaluation of the postmortem application of Bioelectrical Impedance Analysis in a series of 29 human cadavers. By systematically analyzing resistance, reactance, impedance, and phase angle in relation to PMI and BMI across a wide temporal range, and by assessing the effect of autopsy-related anatomical disruption on electrical parameters, this investigation seeks to clarify the feasibility, behavior, and forensic relevance of BIA-derived measurements. The objective of the present exploratory study was to characterize the postmortem behavior of BIA-derived parameters across different PMIs rather than to establish a predictive PMI estimation method.

## Materials and methods

### Study design and forensic setting

The present investigation was conceived as an observational, exploratory pilot study aimed at evaluating the feasibility and postmortem behavior of Bioelectrical Impedance Analysis (BIA) parameters in relation to the postmortem interval (PMI). The study was conducted between September and November 2024 at the Institute of Legal Medicine of the University Magna Graecia of Catanzaro, within the routine medico-legal activity of the institute. All measurements were performed in a *routine* mortuary environment, prior to body refrigeration whenever possible, and in accordance with standard forensic operating procedures. The study was designed as hypothesis-generating, without predefined cut-off values, in order to explore potential correlations between electrical parameters, PMI, and anthropometric variables. The study was intentionally designed as a cross-sectional exploratory analysis based on routine forensic casework rather than a longitudinal repeated-measurement protocol. Repeated serial measurements on the same cadaver were beyond the operational and medico-legal constraints of the present pilot investigation.

### Case selection and inclusion criteria

A total of 29 deceased individuals were consecutively included in the study. Inclusion criteria were the availability of reliable information regarding the time of death, physical accessibility of the body for electrode placement, and the absence of advanced decomposition phenomena that could preclude meaningful bioelectrical measurements. Cases showing severe putrefactive changes, extensive skin loss, or major postmortem trauma interfering with standard electrode positioning were excluded. No restrictions were applied with respect to sex, age, cause of death, or presence of comorbidities, in order to preserve the heterogeneity typical of forensic casework and enhance the external validity of the findings.

### Autopsy status and post-autopsy subgroup

**Fig. 1:**
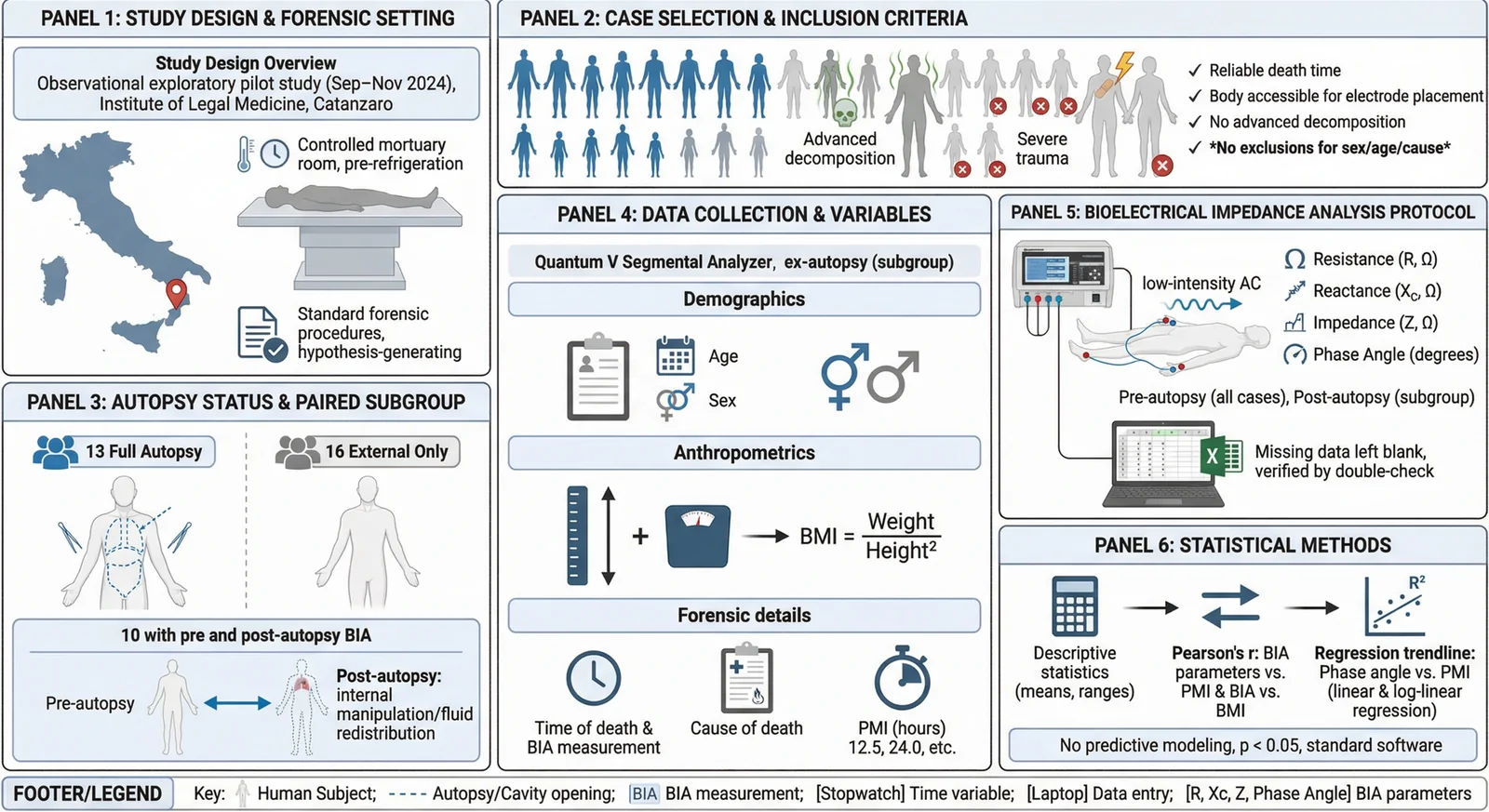
Experimental study design

Of the 29 included cases, 13 underwent full forensic autopsy according to national medico-legal standards, including systematic opening of the cranial, thoracic, and abdominal cavities. Sixteen cases did not undergo autopsy and were examined externally only. In a predefined subgroup of ten autopsied subjects, a second BIA measurement was performed immediately after completion of the autopsy procedure. This subgroup analysis was specifically designed to investigate the potential impact of internal anatomical disruption, organ manipulation, and fluid redistribution induced by autopsy on postmortem bioelectrical parameters (Fig. 1).

### Demographic, anthropometric, and forensic variables

For each subject, demographic data including age and sex were recorded. Anthropometric variables consisted of body weight and height, measured or retrieved from medico-legal documentation, and used to calculate body mass index (BMI) as weight in kilograms divided by height in meters squared. Forensic variables included certified or reconstructed time of death, time of bioelectrical measurement, presumed cause of death, and relevant medical comorbidities when available. Ambient temperature was not systematically recorded during all measurements, although examinations were performed under routine mortuary conditions. The postmortem interval was calculated as the elapsed time between death and BIA assessment and was expressed in decimal hours to ensure analytical precision and consistency in statistical analysis.

### Bioelectrical impedance analysis protocol

Bioelectrical measurements were performed using a Quantum V Segmental Analyzer (RJL Systems, Detroit, MI, USA), a single-frequency device widely validated for clinical body composition analysis. A tetrapolar electrode configuration was adopted, with surface electrodes placed on the right hand and right foot following standard manufacturer recommendations and previously described forensic applications. The bodies were positioned supine on the autopsy table, with limbs slightly abducted to minimize skin contact and electrical interference. A low-intensity alternating current was applied, and measurements were recorded once stable values were obtained. The electrical parameters analyzed included resistance (R, expressed in ohms), reactance (Xc, expressed in ohms), impedance (Z, expressed in ohms), and phase angle (expressed in degrees). All measurements were performed prior to any invasive procedure, except for the post-autopsy recordings, which were obtained using identical electrode placement and device settings.

### Data handling and management

All collected data were entered into a dedicated Microsoft Excel database specifically created for the study. Missing or unavailable data were recorded as blank cells and were not imputed, in order to avoid artificial data distortion. Cases with partial missing data were retained for descriptive analyses but were excluded from specific correlation analyses when relevant variables were unavailable. Data integrity was verified through double-checking of entries against original forensic documentation and device readouts.

### Statistical analysis

Statistical analysis was performed with an exploratory and hypothesis-generating approach. Continuous variables were summarized using descriptive statistics, including mean values and ranges, given the limited sample size and the heterogeneous distribution of the data. The postmortem interval (PMI) was expressed in decimal hours to improve temporal resolution and analytical consistency.

The relationships between PMI and bioelectrical parameters, including resistance, reactance, impedance, and phase angle, were evaluated using Pearson’s correlation coefficient in order to explore the strength and direction of linear associations. Correlation coefficients were interpreted according to conventional criteria, distinguishing weak, moderate, and strong associations. The relationship between PMI and body mass index (BMI) was also examined to assess potential confounding effects of anthropometric variability.

To further characterize the temporal behavior of phase angle, exploratory regression modeling was performed. A simple linear regression model was initially applied to describe the relationship between phase angle and PMI. Given the right-skewed distribution of PMI values and the visual inspection of data dispersion, a logarithmic transformation of PMI was subsequently applied, and a log-linear regression model was constructed with phase angle as the dependent variable and the natural logarithm of PMI as the independent variable. Model performance was evaluated using the coefficient of determination (R²), and residual variability was assessed through calculation of the root mean square error.

Comparative evaluation of bioelectrical measurements obtained before and after forensic autopsy was conducted descriptively in the subgroup of cases with paired measurements, without inferential testing, due to the limited sample size. No multivariate analyses were performed, as the study was not designed to develop predictive models but to explore potential time-dependent trends. Statistical significance was set at p < 0.05, and all analyses were conducted using standard spreadsheet and statistical software.

Given the non-normal distribution and right-skewed nature of PMI values, Spearman’s rank correlation coefficient was additionally calculated to assess monotonic associations independently of normality assumptions.

## Results

The study population consisted of 29 deceased individuals examined over a wide and heterogeneous postmortem interval, allowing evaluation of bioelectrical parameter behavior across different postmortem phases. The postmortem interval ranged from 1.60 to 192.87 h, with a mean value of 38.67 h. The distribution of PMI values was markedly right-skewed, with a higher concentration of cases within the first 48 h after death and a smaller number of subjects examined at longer intervals. This distribution reflects routine forensic casework and allowed exploratory evaluation across different postmortem phases.

Body mass index values showed substantial inter-individual variability, ranging from 9.87 to 57.03 kg/m², with a mean BMI of 26.02 kg/m². The sample therefore included individuals across the full spectrum of nutritional status, from severely underweight to morbidly obese. BMI values were not clustered within specific PMI ranges, and no systematic association between body habitus and time since death was observed at a descriptive level.

Pre-autopsy bioelectrical impedance analysis revealed wide dispersion across all measured electrical parameters. Resistance values ranged from 105.5 to 1053 ohms, with a mean value of 464.77 ohms, indicating pronounced variability in tissue conductive properties among cases. Reactance ranged from 26.9 to 256.9 ohms, with a mean of 83.79 ohms, reflecting heterogeneity in tissue capacitive behavior. Impedance values closely mirrored resistance, ranging from 110.6 to 1073 ohms, with a mean of 472.54 ohms. Phase angle values demonstrated the broadest relative distribution, ranging from 3.3 to 22.4 degrees, with a mean value of 11.24 degrees. The dispersion of phase angle values was evident across the entire PMI range, suggesting that this parameter is influenced by both postmortem temporal factors and inter-individual variability.

Correlation analysis identified distinct relationships between PMI and the evaluated bioelectrical parameters. Phase angle demonstrated a moderate positive association with PMI, with a Pearson correlation coefficient of r = + 0.46 (p = 0.012). The association remained significant when assessed using Spearman’s rank correlation analysis (ρ = +0.58, p = 0.001), indicating persistence of the relationship despite the non-normal distribution of PMI values. Despite considerable variability, phase angle values tended to increase with increasing postmortem interval, although the association remained highly heterogeneous across cases. Reactance showed a weak positive Pearson correlation with PMI (r = + 0.25, p = 0.183), whereas Spearman analysis demonstrated a stronger monotonic association (ρ = +0.52, p = 0.004). Resistance, impedance, and BMI showed no statistically significant associations with PMI in either Pearson or Spearman analyses. In contrast, resistance and impedance showed no meaningful correlation with PMI, with correlation coefficients of r = − 0.07 and r = − 0.05, respectively, indicating that these parameters were largely independent of postmortem time within the studied interval.

The potential influence of body mass index on postmortem electrical behavior was explored by examining the relationship between BMI and PMI. No significant correlation was identified between these variables, although a weak negative trend was observed (*r* = − 0.20).

To further characterize the temporal behavior of phase angle, exploratory regression modeling was performed. A simple linear regression model describing phase angle as a function of PMI demonstrated a positive slope, but with limited explanatory power, accounting for approximately 21% of the observed variance (R² ≈ 0.21). Visual inspection of the data suggested that the relationship between phase angle and PMI was not optimally captured on a linear time scale, particularly at longer PMIs. When PMI was logarithmically transformed, the relationship was better described by a log-linear model, in which phase angle was expressed as a function of the natural logarithm of PMI according to the equation PA = 6.14 + 2.12 · ln(PMI). This model accounted for approximately 38% of the observed variance in phase angle values (R² ≈ 0.38), representing a substantial improvement over the linear model. The regression coefficient for ln(PMI) was statistically significant (p < 0.001), indicating a indicating a potential association between increasing postmortem interval and higher phase angle values. Nevertheless, residual dispersion remained considerable, with a root mean square error of approximately 4.6 degrees, reflecting marked inter-individual variability and limiting the predictive accuracy of the model at the individual case level.

A subgroup analysis was conducted in ten cases for which bioelectrical measurements were obtained both before and after completion of the forensic autopsy. In all examined cases, phase angle values increased following autopsy when compared with pre-autopsy measurements. This increase was observed regardless of PMI duration or BMI category and appeared to be a systematic effect rather than random measurement variability. In contrast, resistance, reactance, and impedance values demonstrated heterogeneous changes after autopsy, with no consistent directional pattern across cases. These findings indicate that invasive postmortem procedures exert a significant influence on phase angle measurements, likely through anatomical disruption, loss of compartmental integrity, and redistribution of fluids.

Taken together, these results indicate that among the evaluated bioelectrical parameters, phase angle shows the most consistent and informative relationship with postmortem interval and follows a time-dependent trend more appropriately described on a logarithmic scale. However, the magnitude of residual variability, the overlap between PMI ranges, and the influence of autopsy-related changes underscore the exploratory nature of these findings and highlight the limitations of using phase angle as a standalone estimator of postmortem interval (Tables 1 and 2).

**Table 1:** Demographic, anthropometric and postmortem characteristics of the study population

Variable	Value
Number of cases	29
Sex	Both sexes
Age (years), range	Adult population
Postmortem interval (hours), range	1.60–192.87
Postmortem interval (hours), mean	38.67
Body mass index (kg/m²), range	9.87–57.03
Body mass index (kg/m²), mean	26.02
Autopsied cases, n (%)	13 (44.8%)
Non-autopsied cases, n (%)	16 (55.2%)
Cases with pre- and post-autopsy BIA	10

PMI is expressed in decimal hours. BMI was calculated as weight (kg)/height² (m²)

**Table 2:** Descriptive statistics of bioelectrical parameters measured before autopsy

Parameter	Mean	Range
Resistance (Ω)	464.77	105.5–1053
Reactance (Ω)	83.79	26.9–256.9
Impedance (Ω)	472.54	110.6–1073
Phase angle (°)	11.24	3.3–22.4

All measurements were obtained prior to any invasive postmortem procedure (Tables 3 and 4).

**Table 3:** Correlation analysis between postmortem interval and bioelectrical parameters

Variable	Pearson’s *r*	*p* value	Spearman’s ρ	*p* value
Resistance	−0.07	0.719	−0.002	0.991
Reactance	+ 0.25	0.183	+ 0.52	0.004
Impedance	−0.05	0.778	+ 0.03	0.893
Phase angle	+ 0.46	0.012	+ 0.58	0.001
Body mass index	−0.20	0.289	−0.22	0.248

**Table 4:** Exploratory regression models describing the relationship between phase angle and postmortem interval

Model	Regression equation	*R*²
Linear	PA = 9.29 + 0.050 · PMI	≈ 0.21
Log-linear	PA = 6.14 + 2.12 · ln(PMI)	≈ 0.38

PA is expressed in degrees and PMI in hours. The log-linear model provided a better fit to the observed data

## Discussion

The present study explored the postmortem application of Bioelectrical Impedance Analysis in a forensic setting and provides novel data on the behavior of bioelectrical parameters in relation to the postmortem interval. The results confirm that BIA is technically feasible in human cadavers under routine medico-legal conditions and that specific electrical parameters exhibit time-dependent changes after death [12–14]. Among the evaluated variables, phase angle showed the strongest observable association with PMI, although the correlation remained moderate and characterized by substantial overlap between different PMIs.

The observed moderate positive correlation between phase angle and PMI represents one of the key findings of this study. This result is particularly relevant because it highlights a divergence between postmortem and in vivo bioelectrical behavior. In clinical settings, phase angle is widely interpreted as a marker of cellular integrity and membrane functionality, with lower values associated with membrane breakdown, fluid imbalance, and adverse outcomes [15–17]. In the postmortem context, however, the biological meaning of phase angle must be reconsidered. After death, the cessation of active cellular processes leads to the progressive loss of transmembrane ion gradients, increased membrane permeability, and redistribution of intra- and extracellular fluids [18]. These changes do not result in an immediate collapse of tissue structure but rather in a gradual transformation of the body into a more electrically homogeneous and passive system. One possible explanation for the observed increase in phase angle is the progressive transition of the body from an actively regulated biological system toward a progressively passive and electrically homogeneous structure. However, this interpretation remains hypothetical and requires dedicated experimental validation. Future studies based on controlled in vitro tissue models and computational simulations of postmortem electrical propagation may help clarify the physical mechanisms underlying these observations.

At present, the biological and physicochemical mechanisms underlying postmortem bioelectrical changes remain insufficiently understood. Consequently, the observed associations should not be interpreted as evidence of a validated thanatochronological mechanism but rather as exploratory observations requiring further mechanistic investigation.

Reactance showed only a weak association with PMI, while resistance and impedance demonstrated no meaningful correlation with postmortem time. These findings suggest that parameters primarily reflecting tissue conductivity and total body water are strongly influenced by inter-individual variability and postmortem fluid shifts, which may obscure time-dependent trends. In contrast, phase angle, which integrates both conductive and capacitive properties, showed a comparatively stronger association with PMI within the limitations of the present dataset. These findings suggest that different bioelectrical parameters may respond differently to postmortem changes and should therefore be interpreted cautiously in forensic settings.

BMI was included only as an exploratory variable potentially influencing bioelectrical measurements. Given the limited sample size and the absence of multivariate modeling, no definitive conclusions regarding the confounding role of BMI can be drawn.

Exploratory regression modeling provided additional insight into the temporal behavior of phase angle. The log-linear relationship observed between phase angle and PMI suggests that postmortem electrical changes may evolve more rapidly during the early postmortem period and progressively stabilize at longer intervals. This pattern is biologically plausible, as early postmortem changes are characterized by rapid ionic redistribution and membrane permeability alterations, followed by slower structural degradation. However, despite improved model fit compared with a simple linear approach, residual variability remained substantial, indicating that phase angle alone cannot provide precise individual-level PMI estimates. Phase angle may provide limited supplementary temporal information; however, the current findings do not support its use as a reliable method for PMI estimation.

An important methodological contribution of this study concerns the effect of forensic autopsy procedures on bioelectrical measurements. In all cases with paired pre- and post-autopsy recordings, phase angle values increased after autopsy, independent of PMI or BMI. This systematic effect suggests that invasive postmortem procedures significantly alter tissue electrical behavior, likely through anatomical disruption, loss of compartmental integrity, and redistribution of fluids. This finding may be relevant for the interpretation of postmortem bioelectrical measurements, as it indicates that post-autopsy BIA measurements are not directly comparable to pre-autopsy values and may introduce substantial bias if measurement timing is not standardized. Consequently, if BIA is to be integrated into forensic practice, measurements should ideally be performed prior to any invasive procedures.

The present findings are consistent with previous reports suggesting postmortem changes in bioelectrical parameters and additionally highlight the confounding effect of autopsy-related manipulation [5–10]. Within the limitations of the present exploratory dataset, phase angle demonstrated the most consistent association with PMI. The exploratory nature of the study and the moderate strength of the observed associations indicate that BIA currently cannot replace established forensic methods and requires substantial additional validation before any practical forensic application can be considered.

Several limitations must be acknowledged. The relatively small sample size restricts statistical power and precludes the development of robust predictive models. Environmental variables such as ambient temperature and humidity were not systematically controlled, and their influence on bioelectrical behavior cannot be excluded. The use of a single-frequency BIA device limits the assessment to global electrical properties and does not allow frequency-dependent analysis of tissue compartments. Nonetheless, these limitations are inherent to pilot forensic studies and do not detract from the methodological and conceptual contributions of the present work. Ambient temperature likely represents a major confounding factor in postmortem bioelectrical behavior, as tissue conductivity, membrane permeability, and fluid redistribution are strongly temperature-dependent phenomena.

The present findings do not support the use of phase angle as a reliable standalone method for PMI estimation. The substantial overlap of values across different PMIs and the marked inter-individual variability indicate that the current approach lacks sufficient discriminative accuracy for forensic application. Rather than validating a new estimation technique, this study primarily demonstrates the complexity and instability of postmortem bioelectrical behavior. The present findings suggest that postmortem BIA parameters exhibit measurable temporal variability; however, the substantial overlap between PMIs currently limits practical forensic applicability.

In conclusion, this exploratory study demonstrates that postmortem bioelectrical parameters, particularly phase angle, undergo measurable changes after death. However, the substantial inter-individual variability, overlap between PMIs, and sensitivity to postmortem manipulations currently preclude reliable forensic application for PMI estimation. The present findings should therefore be interpreted primarily as preliminary observational data highlighting the complexity of postmortem bioelectrical behavior rather than as validation of a novel thanatochronological method.

## References

[1] Lanzinger N, Verhoff MA, Birngruber CG, Lutz L (2026) Factors influencing the progression of post-mortem changes between scene and autopsy. Sci Rep. 10.1038/s41598-026-35786-x 41530243 10.1038/s41598-026-35786-x PMC12804799

[2] Teixeira MJ, Barbosa DJ, Dinis-Oliveira RJ, Freitas AR (2025) Redefining postmortem interval estimation: the need for evidence-based research to bridge science and justice. Front Microbiol 16:1646907. 10.3389/fmicb.2025.1646907 41178973 10.3389/fmicb.2025.1646907 PMC12575334

[3] Obafunwa JO, Roe A, Higley L (2025) Estimating the postmortem interval: applications of forensic acarology, palynology, and taphonomy. Forensic Sci Med Pathol 21(2):940–951. 10.1007/s12024-025-00954-4 39966230 10.1007/s12024-025-00954-4

[4] McIntyre DB, Dawson BM, Long BM, Barton PS (2024) A review of multi-disciplinary decomposition research and key drivers of variation in decay. Int J Legal Med 138(5):2181–2192. 10.1007/s00414-024-03222-2 38622312 10.1007/s00414-024-03222-2 PMC11306653

[5] Bansode S, Morajkar A, Ragade V, More V, Kharat K (2025) Challenges and considerations in forensic entomology: a comprehensive review. J Forensic Leg Med 110:102831. 10.1016/j.jflm.2025.102831 39961182 10.1016/j.jflm.2025.102831

[6] Hansen ES, Baigent C, Reck SI, Connor M (2017) Bioelectrical impedance as a technique for estimating postmortem interval. J Forensic Sci 63(4):1186–1190. 10.1111/1556-4029.13695 29143324 10.1111/1556-4029.13695

[7] Nishida A, Kim WC, Yoshida T, Oka Y, Yamada N, Nakase M et al (2015) A new method for the estimation of age at death by using electrical impedance: a preliminary study. Leg Med (Tokyo) 17(6):560–568. 10.1016/j.legalmed.2015.07.003 26162996 10.1016/j.legalmed.2015.07.003

[8] Ellis KJ, Bell SJ, Chertow GM, Chumlea WC, Knox TA, Kotler DP et al (1999) Bioelectrical impedance methods in clinical research: a follow-up to the NIH technology assessment conference. Nutrition 15:874–880. 10.1016/S0899-9007(99)00147-1 10575664 10.1016/s0899-9007(99)00147-1

[9] Stahn A, Terblanche E, Gunga HC (2012) Use of bioelectrical impedance: general principles and overview. In: Preedy VR (ed) Handbook of Anthropometry. Springer, New York, pp 49–90. doi:10.1007/978-1-4419-1788-1_3

[10] Lackermair K, Fischer F, Manhart J, Scheurer E, Graw M, Boy D et al (2022) Determination of time of death by blinded post-mortem interrogation of cardiac implantable electrical devices. Sci Rep. 10.1038/s41598-022-12390-3 35581374 10.1038/s41598-022-12390-3 PMC9112646

[11] Singh J, Kumar A, Trivedi S, Pandey SK (2025) Advancements in estimating post-mortem interval in medico-legal practice: a comprehensive review. Leg Med (Tokyo) 75:102627. 10.1016/j.legalmed.2025.102627 40273647 10.1016/j.legalmed.2025.102627

[12] Norman K, Stobäus N, Pirlich M, Bosy-Westphal A (2012) Bioelectrical phase angle and impedance vector analysis: clinical relevance and applicability of impedance parameters. Clin Nutr 31(6):854–861. 10.1016/j.clnu.2012.05.008 22698802 10.1016/j.clnu.2012.05.008

[13] Garlini L, Alves FD, Ceretta LB, Perry IDS, Souza GC, Clausell N (2019) Phase angle and mortality: a systematic review. Eur J Clin Nutr 73:495–508. 10.1038/s41430-018-0159-1 29695763 10.1038/s41430-018-0159-1

[14] Popiołek-Kalisz J, Kalisz G, Zembala M (2025) The application of bioelectrical impedance analysis phase angle in cardiac surgery. Nutrients 17:1914. 10.3390/nu17111914 40507183 10.3390/nu17111914 PMC12158013

[15] Lima J, Eckert I, Gonzalez MC, Silva FM (2022) Prognostic value of phase angle and bioelectrical impedance vector in critically ill patients: a systematic review and meta-analysis of observational studies. Clin Nutr 41(12):2801–2816. 10.1016/j.clnu.2022.10.010 36395589 10.1016/j.clnu.2022.10.010

[16] Ward LC, Brantlov S (2023) Bioimpedance basics and phase angle fundamentals. Rev Endocr Metab Disord 24:381–391. 10.1007/s11154-022-09780-3 36749540 10.1007/s11154-022-09780-3 PMC10140124

[17] Lee G, Kim E (2023) Usefulness of phase angle on bioelectrical impedance analysis as a surveillance tool for postoperative infection in critically ill patients. Front Med (Lausanne) 10:1111727. 10.3389/fmed.2023.1111727 36910475 10.3389/fmed.2023.1111727 PMC9992789

[18] Sacco MA, Cordasco F, Scalise C, Ricci P, Aquila I (2022) Systematic review on post-mortem protein alterations: analysis of experimental models and evaluation of potential biomarkers of time of death. Diagnostics (Basel) 12(6):1490. 10.3390/diagnostics12061490 35741301 10.3390/diagnostics12061490 PMC9222196

